# Maple compounds prevent biofilm formation in *Listeria monocytogenes* via sortase inhibition

**DOI:** 10.3389/fmicb.2024.1436476

**Published:** 2024-09-16

**Authors:** Ahmed M. Elbakush, Oliver Trunschke, Sulman Shafeeq, Ute Römling, Mark Gomelsky

**Affiliations:** ^1^Department of Molecular Biology, University of Wyoming, Laramie, WY, United States; ^2^Department of Microbiology, Tumor and Cell Biology (MTC), Karolinska Institutet, Stockholm, Sweden

**Keywords:** biofilm, *Listeria (L.) monocytogenes*, antibiofilm, sortase A inhibitor, maple, exopolysaccharide, surface attachment, fresh produce and foodborne illness

## Abstract

The Pss exopolysaccharide (EPS) enhances the ability of the foodborne pathogen *Listeria monocytogenes* to colonize and persist on surfaces of fresh fruits and vegetables. Eradicating listeria within EPS-rich biofilms is challenging due to their increased tolerance to disinfectants, desiccation, and other stressors. Recently, we discovered that extracts of maple wood, including maple sap, are a potent source of antibiofilm agents. Maple lignans, such as nortrachelogenin-8’-O-β-D-glucopyranoside and lariciresinol, were found to inhibit the formation of, and promote the dispersion of pre-formed *L. monocytogenes* EPS biofilms. However, the mechanism remained unknown. Here, we report that these lignans do not affect Pss EPS synthesis or degradation. Instead, they promote EPS detachment, likely by interfering with an unidentified lectin that keeps EPS attached to the cell surfaces. Furthermore, the maple lignans inhibit the activity of *L. monocytogenes* sortase A (SrtA) *in vitro*. SrtA is a transpeptidase that covalently anchors surface proteins, including the Pss-specific lectin, to the cell wall peptidoglycan. Consistent with this, deletion of the *srtA* gene results in Pss EPS detachment from listerial cells. We also identified several additional maple compounds, including epicatechin gallate, isoscopoletin, scopoletin, and abscisic acid, which inhibit *L. monocytogenes* SrtA activity *in vitro* and prevent biofilm formation. Molecular modelling indicates that, despite their structural diversity, these compounds preferentially bind to the SrtA active site. Since maple products are abundant and safe for consumption, our finding that they prevent biofilm formation in *L. monocytogenes* offers a viable source for protecting fresh produce from this foodborne pathogen.

## Introduction

Fresh produce contaminated with the bacterial foodborne pathogen *Listeria monocytogenes* has been linked to several listeriosis outbreaks in recent decades, rivaling traditional sources of contamination such as deli meat, poultry, fish, and dairy products (Zhu et al., 2017). According to the Centers for Disease Control and Prevention (CDC), recent outbreaks in the USA have originated from contaminated whole cantaloupes (rock melons), frozen vegetables, cut celery, packaged salads, bean sprouts, caramelized apples, and mushrooms [Centers for Disease Control and Prevention (CDC), 2022]. In otherwise healthy individuals, listerial infections typically remain confined to the gastrointestinal tract and are rarely life-threatening. However, in individuals with compromised immune systems, the elderly, pregnant women, fetuses, and young children, these infections can progress to systemic listeriosis. The mortality rates for those who do develop listeriosis are alarmingly high, reaching 15–20% in Western countries. As a result, strict regulations are in place regarding acceptable levels of *L. monocytogenes* in ready-to-eat food products. In the USA, there is a “zero tolerance” policy (Archer, 2018), while the European Union allows less than 100 colony-forming units (CFUs) per 100 grams of food (European Commission, 2005). Frequent product recalls due to confirmed or suspected contamination make *L. monocytogenes* a costly foodborne pathogen (Hoffmann and Ahn, 2021).

Completely avoiding contamination of fresh produce is challenging, if not impossible, because *L. monocytogenes* is ubiquitous in the environment (Marik et al., 2020). Additionally, the relative importance of various contamination sources is not fully understood. For instance, a recent metagenomic analysis revealed that significant percentages of both farm animals and, surprisingly, humans may carry *L. monocytogenes* asymptomatically (Hafner et al., 2021). However, in most cases, fresh produce contamination occurs postharvest, particularly at storage and processing facilities (Ferreira et al., 2014; Garner and Kathariou, 2016; Rodríguez-López et al., 2018; Marik et al., 2020). The cleaning and disinfection protocols at these facilities often fail to eliminate *L. monocytogenes*, especially if biofilms are present on the fresh produce or in hard-to-reach areas of processing equipment. To effectively eradicate *L. monocytogenes* biofilms, more than just cleaning and disinfection may be required; specific antibiofilm agents may need to be applied (Fagerlund et al., 2020).

The Pss exopolysaccharide (EPS) is a recently identified component of listerial biofilms that plays a crucial role in their resilience. *L. monocytogenes* produces Pss when intracellular levels of c-di-GMP are elevated (Chen et al., 2014; Köseoğlu et al., 2015). C-di-GMP, a second messenger, activates EPS synthesis and promotes biofilm formation in various bacteria (Römling et al., 2013; Poulin and Kuperman, 2021). The Pss EPS enhances the colonization of plant surfaces, including fruits and vegetables, but does not significantly improve colonization of manmade materials. Strains that overproduce Pss colonize rough plant surfaces, such as cantaloupe rind, more than 10-fold more efficiently than strains with impaired Pss synthesis. On smooth surfaces, such as cantaloupe flesh, the difference is less pronounced, approximately 2-fold (Fulano et al., 2023). Importantly, bacteria within Pss-containing biofilms exhibit greater tolerance to desiccation, disinfectants, and hydrochloric acid (Chen et al., 2014; Fulano et al., 2023). These stresses are particularly relevant to the storage, transportation, and consumption of fresh produce (Tennant et al., 2008; Esbelin et al., 2018). Consequently, Pss EPS-synthesizing strains have a significant advantage, approximately 10^2^ to 10^4^ times greater, in reaching the small intestines of consumers, which are the primary infection sites for this pathogen (Fulano et al., 2023). This highlights Pss EPS as a risk factor for fresh produce safety.

The Pss EPS has a distinct composition and structure. It consists of a chain of N-acetyl mannosamine (ManNAc) disaccharide units linked by (1–4)-β-glycosidic bonds, with every other ManNAc residue decorated with α-galactose {4)-β-ManpNAc-(1–4)-[α-Galp-(1–6)]-β-ManpNAc-(1-} (Köseoğlu et al., 2015). The significance of Pss EPS for the survival of *Listeria* in the environment is underscored by the fact that the *pss* operon, which encodes the Pss biosynthetic machinery (Chen et al., 2014), is part of the core genome of *L. monocytogenes*, as revealed by the genomes of over a thousand sequenced isolates (Moura et al., 2016). The high conservation of the *pss* operon among nonpathogenic *Listeria* species (Chen et al., 2014) further emphasizes its importance for environmental survival.

Recently, we discovered that aqueous extracts from maple wood, including maple sap and syrup, have strong anti-EPS properties. For instance, dilutions of commercially available maple syrup at 1:200 or higher effectively prevented biofilm formation by Pss-overproducing strains on various fruits and vegetables, and caused the dispersion of existing EPS biofilms. We found that two lignans in maple wood products — nortrachelogenin-8’-O-β-D-glucopyranoside (NTG) (Wan et al., 2012) and lariciresinol (LR) (St-Pierre et al., 2014) — are responsible for this anti-EPS activity (Elbakush et al., 2023). However, the exact mechanism by which these compounds work has remained unknown. Elucidating such a mechanism has been the major goal of this study. Additionally, we identified several non-lignan antibiofilm compounds in maple. Given the abundance, affordability, and lack of toxicity of maple products, along with the newly discovered antibiofilm mechanism, there is great potential for their use in protecting fresh produce from listerial contamination.

## Results

### Assessing the spectrum of antibiofilm activity of maple compounds

Before investigating the mechanism of action of maple lignans, we assessed whether their antibiofilm activity extends to other fresh produce pathogens beyond *L. monocytogenes*. Specifically, we tested the impact of maple compounds on biofilm formation by another common foodborne pathogen, *Salmonella enterica* subsp. Typhimurium. Unlike *L. monocytogenes*, a monoderm belonging to the Bacillota (Firmicutes) phylum, *S. typhimurium* is a diderm gammaproteobacterium from the Pseudomonadota (Proteobacteria) phylum.

**Figure 1:**
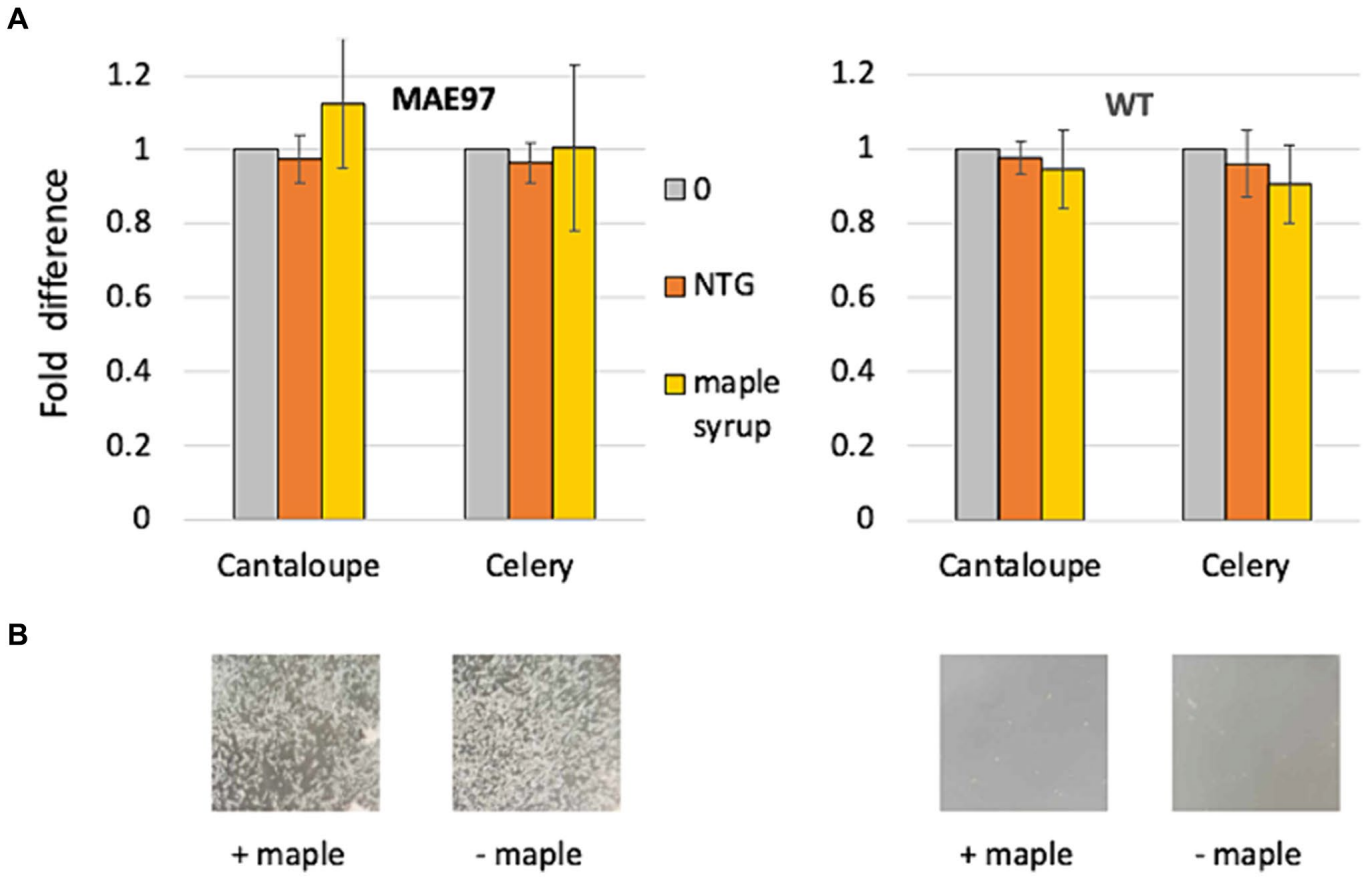
Maple compounds do not inhibit colonization of fresh produce by *S. typhimurium*. Standard cantaloupe rind-containing round coupons and pieces of cut celery were incubated in liquid HTM/G medium for 48 h at 30°C in the presence *S. typhimurium* strains, MAE97 (high c-di-GMP strain) and UMR1 (wild type). The following compounds were added at the time of inoculation -- NTG (120 μM, final concentration), maple syrup (1:200 dilution), or DMSO (0, negative control). The numbers of attached bacteria were counted following rinsing and maceration in PBS of the cantaloupe and celery biomass, and plating of diluted suspension on LB. **(A)** Fold-difference in CFUs of *S. typhimurium* attached to the cantaloupe rind-containing coupons and celery pieces. CFUs in the presence of DMSO were set at 1. Results of two experiments, each involving three produce pieces. **(B)** Representative images of the appearance of bacterial cultures incubated with DMSO (“− maple”) and maple syrup (“+ maple”) at the end of 48-h incubation. Note that clumps formed by the high c-di-GMP strain are not affected by the presence of maple syrup.

We incubated *S. typhimurium* strains in minimal liquid media (HTM/G) with sterile pieces of cantaloupe rind or cut celery under the same conditions used in recent experiments with *L. monocytogenes* (Elbakush et al., 2023). The high c-di-GMP strain MAE97 overproduces solely the primary *S. typhimurium* EPS, phosphoethanolamine-modified cellulose (PEAC) (Zogaj et al., 2001; Yaron and Römling, 2014; Thongsomboon et al., 2018). Similar to the Pss-overproducing *L. monocytogenes* strain, the PEAC-overproducing strain formed clumps in the HTM/G medium (Figure 1A). However, neither diluted maple syrup nor the lignan NTG prevented PEAC-mediated cell aggregation in this strain (Figure 1A). Consistent with the lack of antibiofilm activity against *S. typhimurium*, there was no significant reduction in the CFUs of *S. typhimurium* attached to cantaloupe or cut celery surfaces, whether in the PEAC overexpressing strain, MAE97, or the wild type strain, UMR1 (Figure 1B). Furthermore, maple wood extracts did not reduce biofilm formation of *S. typhimurium* on polystyrene microtiter plates in rich LB medium lacking NaCI (Supplementary Figure S1). These findings suggest that the antibiofilm activity of maple compounds is specific to *L. monocytogenes* or potentially to other members of the Bacillota phylum.

### Search for the target of the antibiofilm activity of maple lignans

**Figure 2:**
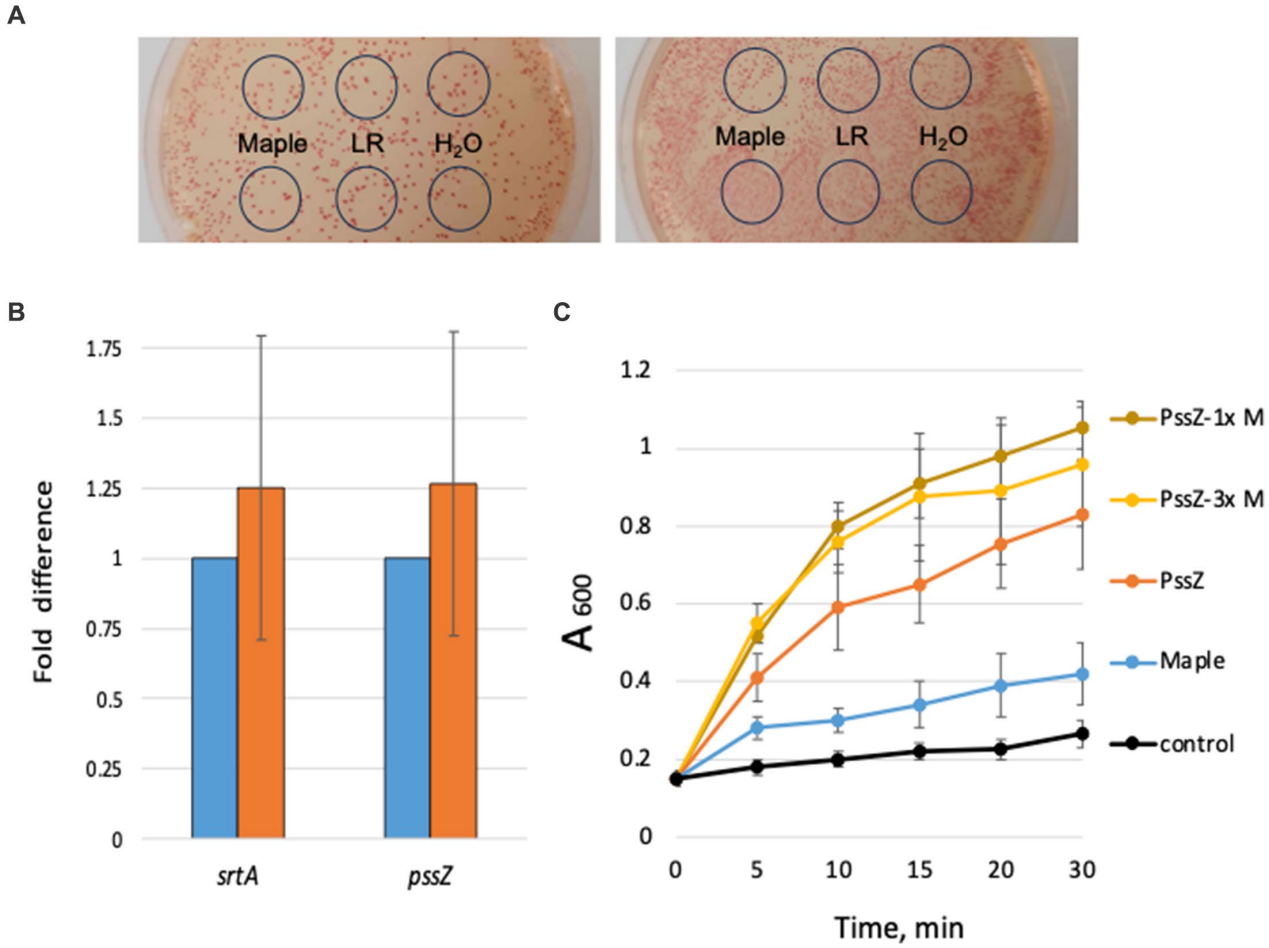
Maple compounds affect neither Pss synthesis nor hydrolysis. **(A)** Maple compounds do not appear to affect Pss EPS synthesis. The high c-di-GMP strain, ∆*pdeB/C/D*, was plated on the HTM/G agar medium containing Congo red dye. Two representative plates have a 10-fold difference in colony numbers. Five μL of diluted (1:200) maple syrup (“Maple”), 80 μM LR solution, or H_2_O were spotted inside the marked circles after plating. The Congo red staining of colonies does not differ in the presence of maple compounds. **(B)** Maple compounds do not significantly affect abundance of the *pssZ* or *srtA* mRNA. Results of qRT-PCR based on RNA purified from the ∆*pdeB/C/D* strain (light-blue bars; value set at 1.0) and ∆*pdeB/C/D* grown in the presence of 1:200 maple syrup (orange bars). **(C)** Maple compounds do not accelerate PssZ-mediated Pss hydrolysis. Clump dispersion assay was performed as described earlier (Elbakush et al., 2023). Control, HTM/G medium; PssZ, 0.13 μg mL^−1^ PssZ::His6; Maple, 1:200 maple syrup; PssZ-1x, PssZ plus 1:200 maple syrup; PssZ-3x, PssZ plus 1:70 maple syrup.

In investigating the potential mechanism behind the antibiofilm activity of maple lignans in *L. monocytogenes*, we initially considered two possibilities: (i) inhibition of Pss synthesis or (ii) activation of Pss hydrolysis. To test the effect on Pss synthesis, we used a Congo red assay. Congo red is a dye that stains various types of EPS, including Pss (Chen et al., 2014). We observed that colonies of the high c-di-GMP strain, *ΔpdeB/C/D*, grown with diluted maple syrup, LR (Figure 2A), or NTG (not shown) retained their red color, similar to colonies grown without these compounds. This indicates that Pss synthesis is largely unaffected by the maple compounds.

We then explored whether maple compounds might activate Pss hydrolysis, leading to smaller Pss fragments that do not support cell adhesion. To this end, we measured the impact of diluted maple syrup on the abundance and activity of the Pss hydrolase, PssZ, known to degrade Pss EPS (Köseoğlu et al., 2015). Due to the lack of PssZ-specific antibodies, we assessed *pssZ* transcript levels using quantitative RT-PCR. The maple lignans did not significantly increase *pssZ* mRNA (Figure 2B), suggesting that they are unlikely to drastically elevate PssZ abundance.

To determine if maple syrup enhances PssZ activity, we conducted a clump dispersion assay. This assay monitors the release of bacteria from preformed clumps by measuring the increase in absorbance of bacterial suspensions over time after the undispersed clumps settle (Köseoğlu et al., 2015; Elbakush et al., 2023). If maple compounds activated PssZ, we would expect faster dispersion in the presence of maple syrup. However, we found that the addition of diluted maple syrup resulted in only modest, immediate clump dispersion (Figure 2C), with no significant change over the 30-min experimental period. This suggests that maple compounds do not enhance PssZ activity. Note that we could not use a *pssZ* null mutant as a control because this mutant is impaired in Pss production (Köseoğlu et al., 2015).

To further investigate the hypothesis that maple compounds activate PssZ-dependent Pss hydrolysis, we measured clump dispersion with the addition of purified PssZ protein (Köseoğlu et al., 2015). As expected, the addition of exogenous PssZ promoted clump dispersion. However, when PssZ was combined with maple syrup, clump dispersion was not accelerated (Figure 2C, PssZ versus PssZ-1x). This suggests that maple compounds do not activate PssZ. Increasing the concentration of maple syrup threefold also failed to enhance dispersion (Figure 2C, PssZ-1x versus PssZ-3x), reinforcing our conclusion. Similar results were obtained with NTG instead of maple syrup, leading us to reject the hypothesis that maple compounds activate PssZ-mediated Pss hydrolysis.

**Figure 3:**
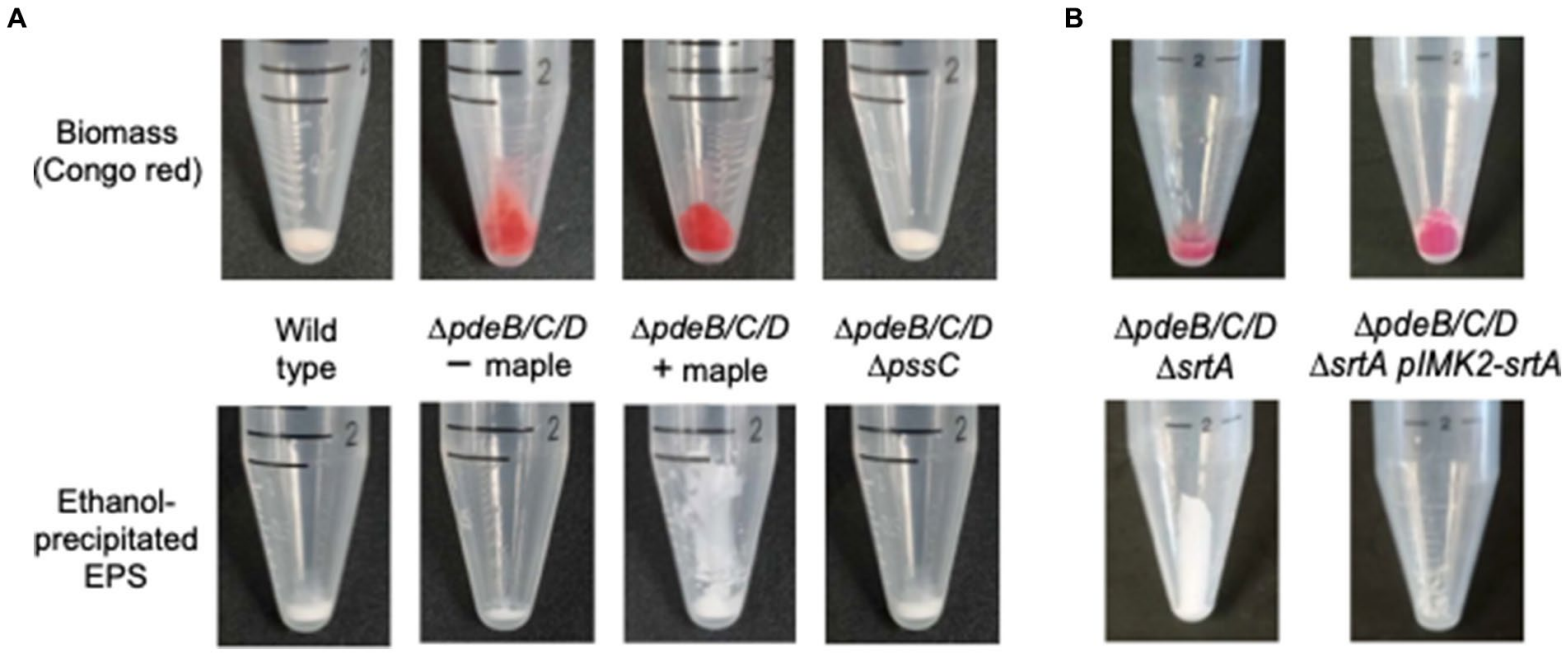
Maple lignans induce Pss detachment from bacterial cell surfaces. **(A)** The biomass and ethanol-precipitated EPS from *L. monocytogenes* wild type, EGD-e, and its high-c-di-GMP derivative, ∆*pdeB/C/D*, grown in the presence (“+ maple”) or absence (“- maple”) of maple compounds. Congo red was added to stain the EPS associated with the biomass. **(B)** Congo red stained biomass and ethanol-precipitated EPS from the *srtA* mutant of the high c-di-GMP strain, *∆pdeB/C/D ∆srtA*, and the *∆pdeB/C/D ∆srtA* strain complemented by the chromosome-intgrated intact *srtA* gene.

Given that maple compounds did not affect Pss synthesis or degradation, we explored the possibility that they influence the attachment of Pss EPS to bacterial cells. If attachment were impaired, we would expect Pss to accumulate in the supernatant of liquid cultures, while remaining within bacterial colonies on solid media, which would explain the red colony appearance in the presence of Congo red dye (Figure 2A). To test this, we grew the high c-di-GMP strain in liquid medium with and without maple syrup, then centrifuged the bacterial biomass and precipitated the soluble extracellular EPS from the supernatant using cold ethanol (Köseoğlu et al., 2015). Consistent with our hypothesis, the amount of ethanol-precipitated material from the *ΔpdeB/C/D* strain grown with maple syrup was significantly higher than without (Figure 3A). Both the wild type and the *ΔpdeB/C/D ΔpssC* mutant, which do not synthesize Pss (Chen et al., 2014), produced no cell-attached EPS, as indicated by the absence of Congo red staining of their biomass (Figure 3A). The small amount of precipitated soluble EPS in these strains likely represents lipoteichoic acids shed from the cell surfaces, as previously shown (Köseoğlu et al., 2015).

### Maple lignans target the *Listeria monocytogenes* sortase SrtA

The detachment of Pss from bacterial cell surfaces induced by maple compounds could be explained by two potential mechanisms: (i) these compounds may interfere with Pss EPS binding to the Pss-specific lectin on the surface of listeria, or (ii) they may reduce the abundance of the lectin on cell surfaces. Given that the identity of the Pss-specific lectin is currently unknown, we focused on the second possibility. Most surface proteins in *L. monocytogenes* are covalently anchored to the cell wall peptidoglycan by transpeptidases known as sortases (Schneewind and Missiakas, 2014). If maple compounds inhibit sortase activity or reduce sortase levels, the abundance of the Pss-specific lectin on the cell surface could be decreased.

*L. monocytogenes* has two sortases: SrtA, which anchors the majority of surface proteins, and SrtB, which anchors proteins involved in heme uptake (Bierne et al., 2002; Bierne et al., 2004). If SrtA were targeted by maple lignans, a *ΔsrtA* deletion in the high c-di-GMP strain would be expected to mimic the effect of maple syrup on Pss detachment. Indeed, the constructed here *ΔpdeB/C/D ΔsrtA* mutant produced significantly more soluble EPS, resembling the effect of maple compounds (Figure 3B). To complement the *ΔsrtA* mutation, we inserted the intact *srtA* gene into the chromosome of the *ΔpdeB/C/D ΔsrtA* strain via the integrative vector pIMK2. Complementation resulted in the restoration the mutant *ΔpdeB/C/D* phenotype, i.e., retention of the Pss EPS on listerial cells (Figure 3B).

**Figure 4:**
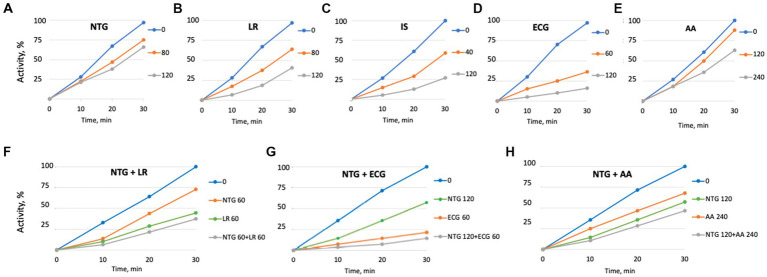
Sortase A is the target of maple lignans. SrtA inhibition by various maple compounds. Activity of the purified membrane-less SrtA::His_6_ was tested using the fluorescently labelled LPxTG-containing peptide substrates. **(A)** nortrachelogenin-8’-O-β-D-glucoside (NTG); **(B)** lariciresinol (LR); **(C)** isoscopoletin (IS); **(D)** (−)-epicatechin gallate (ECG); **(E)** abscisic acid (AA); **(F)** NTG plus LR; **(G)** NTG plus ECG; **(H)** NTG plus AA. Concentrations (in μM) are shown in the legends. The average value of two experiments is displayed. Standard deviations did not exceed 14% of the average values.

We then investigated whether maple lignans affect the abundance or activity of SrtA. Quantitative RT-PCR analysis showed no significant differences in *srtA* mRNA levels in cultures grown with or without maple compounds (Figure 2B), suggesting that maple compounds do not drastically reduce SrtA protein levels. To assess the inhibitory effects of maple lignans on SrtA, we purified the truncated SrtA protein that lacks the N-terminal membrane-spanning domain (Li et al., 2016). The peptidase activity of SrtA was measured by monitoring the proteolysis of a fluorescently labeled peptide containing the SrtA-specific LPxTG motif. As shown in Figure 4A, both maple lignans, NTG and LR, moderately inhibited SrtA activity in a dose-dependent manner, indicating that SrtA is a direct target of the maple lignans.

### Multiple maple compounds inhibit *Listeria monocytogenes* SrtA

While the inhibition of SrtA activity *in vitro* by NTG and LR aligns with their observed antibiofilm effects, an intriguing discrepancy remained: diluted maple syrup exhibited greater antibiofilm potency than anticipated. Specifically, a 1:200 dilution of maple syrup should not contain lignan concentrations in the ten-to-hundred micromolar range used in the *in vitro* assays. For instance, the concentration of LR in undiluted maple syrup has been reported to be approximately 54 μM (St-Pierre et al., 2014). After a 1:200 dilution, the LR concentration would drop to the sub-micromolar range.

To resolve this discrepancy, we hypothesized that additional compounds in maple syrup might also inhibit SrtA and that their effectiveness could surpass that of the lignans. We tested several polyphenolic compounds known to be present in maple syrup and maple wood extracts (Ramadan et al., 2021; Wan et al., 2012). Among these, we identified several compounds with potent anti-SrtA activities. (−)-Epicatechin gallate (ECG), isoscopoletin (IS), and scopoletin showed stronger SrtA inhibition *in vitro* than NTG and LR (Figures 4A–D), while abscisic acid (AA), which is relatively abundant in maple syrup (Ramadan et al., 2021), exhibited lower SrtA inhibitory activity (Figure 4E). Interestingly, clump dispersion by ECG is more effective at lower concentrations (30–60 μM) than the concentration (120 μM) originally used for screening of maple compounds (Elbakush et al., 2023). This explains why ECG was not prioritized by us earlier, though the reason for this unexpected phenomenon is not immediately clear.

**Figure 5:**
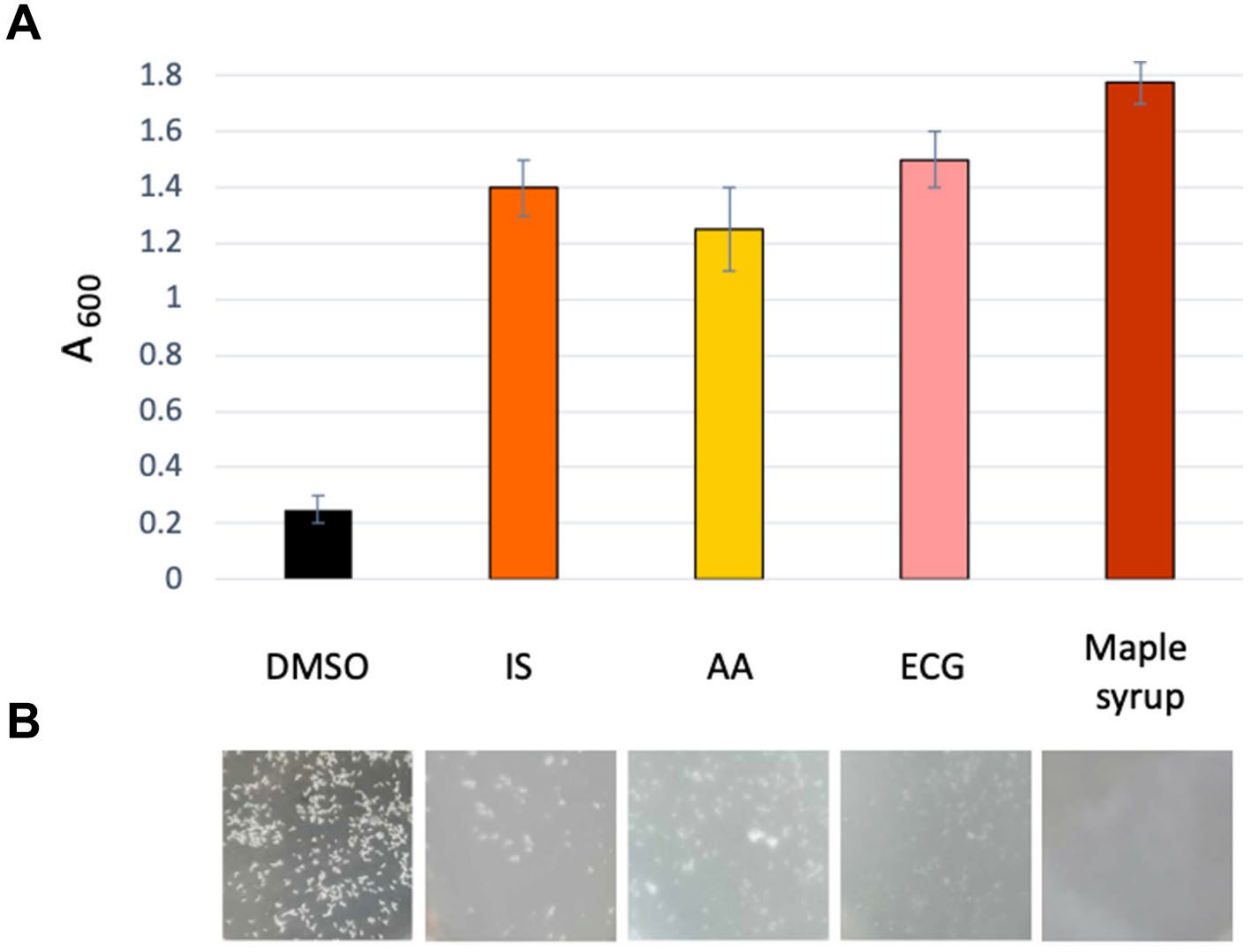
Newly identified maple compounds with anti-SrtA activity inhibit EPS formation. **(A)** Dispersion of EPS-clumps of the *L. monocytogenes* high-c-di-GMP strain, ∆*pdeB/C/D*, by the newly identified SrtA inhibitors from maple. Absorbance (A_600_) of bacterial culture is measured at the end of the 48-h incubation in the presence of the indicated compounds. A_600_ was measured after a 2-min pause needed for spontaneous precipitation of undispersed clumps (Elbakush et al., 2023). The higher the absorbance the more potent dispersion. **(B)** Representative images of the appearance of bacterial cultures after 48-h incubation in the presence of the maple compounds or 1:200 diluted maple syrup.

We next assessed whether the anti-SrtA activity of these newly identified maple compounds correlated with their ability to inhibit listerial EPS-biofilms. We inoculated the high c-di-GMP strain in the presence of 60 μM of each compound and measured antibiofilm activity using the clump dispersion assay (Elbakush et al., 2023). As shown in Figures 5A,B, all three compounds effectively inhibited clumping. These findings suggest that the high potency of maple extracts as listerial antibiofilm agents is largely attributable to the presence of several SrtA inhibitors.

### SrtA inhibitory activity *in vitro* does not necessarily correlate with antibiofilm activity

To further investigate whether *in vitro* SrtA inhibitory activity correlates with anti-EPS effects, we tested previously identified SrtA inhibitors of *L. monocytogenes*: genistin (Liu et al., 2023), chalcone (Li et al., 2016), and baicalein (Lu et al., 2019) at concentrations of 60 and 120 μM. None of these compounds prevented listerial clump formation. Notably, chalcone was found to be insoluble and precipitated out of the growth medium (Supplementary Figure S2A).

We also evaluated several inhibitors of SrtA enzymes from other pathogens of the Bacillota (Firmicutes) phylum that had not been previously tested against *L. monocytogenes* SrtA. These included astibin (Wang et al., 2019), curcumin (Hu et al., 2013), and morin (Kang et al., 2006; Huang et al., 2014). All these compounds were ineffective in preventing *L. monocytogenes* EPS-biofilm formation (Supplementary Figure S2B). These results indicate that *in vitro* SrtA inhibitory activity does not necessarily translate to protection against listerial EPS-biofilms.

### Molecular modelling of the *Listeria monocytogenes* SrtA interactions with maple compounds

**Figure 6:**
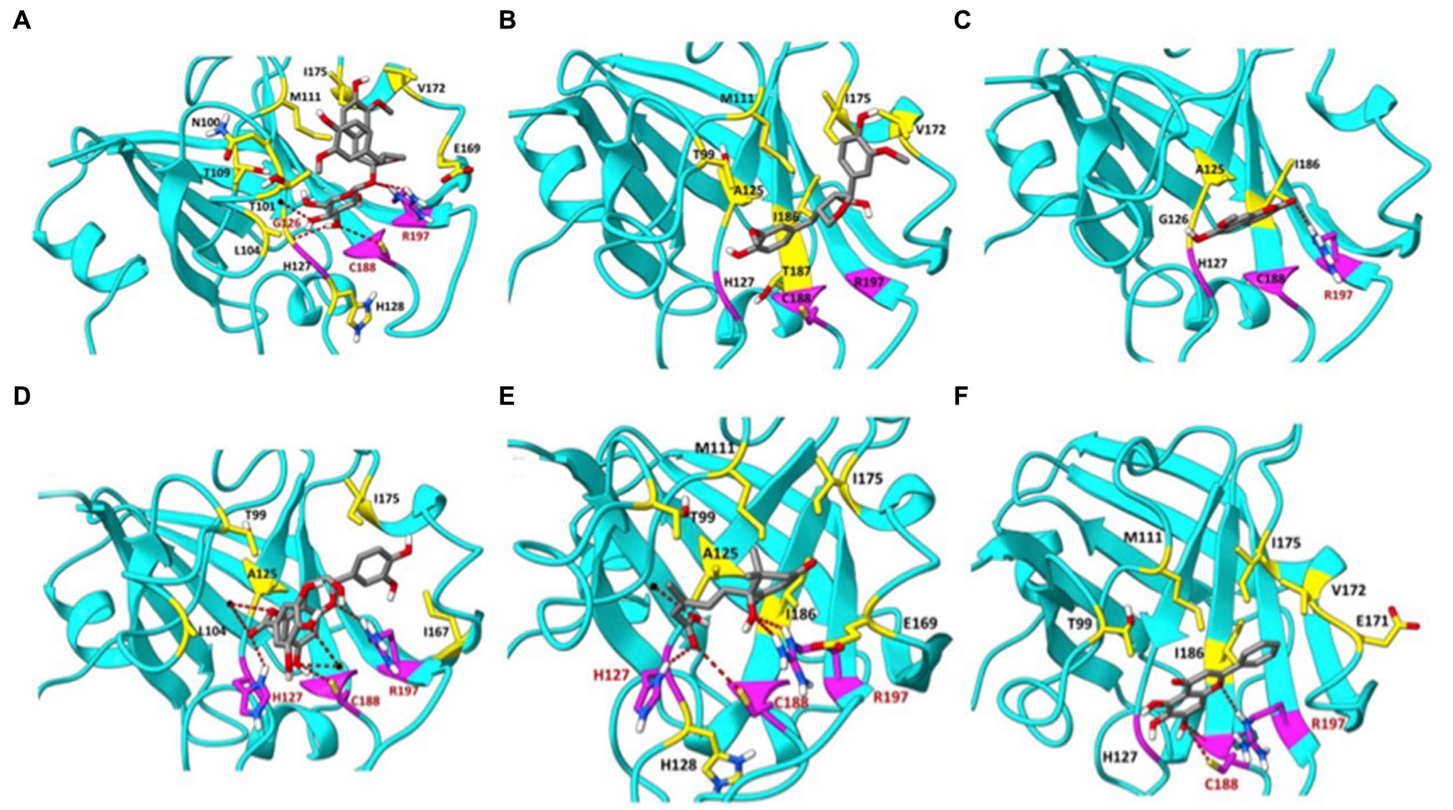
Molecular modelling of the SrtA interactions with selective maple compounds. The X-ray structure of the *L. monocytogenes* SrtA (PDB: 5HU4) was used to predict binding modes of the maple compounds using the Autodoc Vina software (Trott and Olson, 2010). The predicted binding sites and chemical bonds are shown on the panels corresponding to individual compounds. Grid Box measurements were established with AutoDockTools-1.5.7. Box coordinates: X: 5.117 Å, Y: 20.439 Å, Z: 11.52 Å; Box Size: X: 52 Å, Y: 58 Å, Z: 58 Å, with default spacing 0.375, Mode Number: 3, Energy Range: 5, Exhaustiveness: 8. The amino acid residues of the catalytic triad of SrtA (H127, C188, R197) are colored in magenta. H-bonds are shown in red. Amino acids forming hydrophobic interactions with maple compounds are shown in yellow, except for hydrophobic interactions with the amino acids of the catalytic triad, which are shown in black. **(A)** nortrachelogenin-8’-O-β-D-glucoside (NTG); **(B)** lariciresinol (LR); **(C)** isoscopoletin (IS); **(D)** (−)-epicatechin gallate (ECG); **(E)** abscisic acid (AA); **(F)** baicalein.

The maple compounds that inhibit SrtA belong to various chemical classes: NTG and LR are lignans, ECG is a catechin, IS is a coumarin, and AA is an abscisic acid derivative. The effectiveness of these structurally diverse compounds in inhibiting *L. monocytogenes* SrtA is somewhat surprising. To understand how these compounds might inhibit SrtA, we conducted *in silico* modelling using the X-ray structure of *L. monocytogenes* SrtA (Li et al., 2016) and the protein-ligand docking software Autodock Vina (Trott and Olson, 2010). Our molecular models indicated that all maple compounds with anti-SrtA activity bind within the catalytic site of SrtA (Figures 6A–E) with negative ∆G values ranging from-5.0 to-7.4 kcal/mol (Table 1). The most potent SrtA inhibitor *in vitro*, ECG, has the most negative ∆G value and is predicted to form hydrogen bonds with each of the catalytic triad residues of SrtA: H127, C188, and R197 (Li et al., 2016) (Figure 6E). Notably, baicalein, another SrtA inhibitor, has a ∆G value similar to that of ECG (Table 1; Figure 6F). However, the ∆G values of other maple compounds did not directly correlate with their potency as SrtA inhibitors *in vitro* or their antibiofilm activity *in vivo*. For instance, the second most potent SrtA inhibitor and antibiofilm agent, IS (Figures 4A, 5), has a lower absolute ∆G value compared to other antibiofilm compounds (Table 1). Like baicalein (Figure 6F) and LR (Figure 6B), IS forms mostly hydrophobic interactions within the catalytic site (Figure 6C). In contrast, AA has a better ∆G value than IS (Table 1) and forms hydrogen bonds with each residue of the SrtA catalytic triad (Figure 6E), yet it is less effective as an SrtA inhibitor *in vitro* and *in vivo* compared to IS (Figures 4A, 5).

**Table 1:** Predicted ΔG of binding of SrtA inhibitors.

Compound	∆G, kcal/mol
Nortrachelogenin-8’-O-β-D-glucoside (NTG)	−7.2
Lariciresinol (LR)	−6.2
Isoscopoletin (IS)	−5.0
(−)-Epicatechin gallate (ECG)	−7.4
Abscisic acid (AA)	−5.9
Baicalein	−7.4

Since the *in silico* models predict that all inhibitors bind within the catalytic site, adding two inhibitors at nonsaturating concentrations was expected to have an additive effect. We tested this by examining selected inhibitor pairs and observed additive inhibitory effects (Figures 4F–H). This finding does not preclude the possibility of synergistic effects with other combinations of SrtA inhibitors or with combinations of inhibitors and non-inhibitory maple compounds. Interestingly, some SrtA inhibitors might bind to secondary sites outside the catalytic site (Supplementary Figure S3). These peripheral interactions could potentially impair SrtA’s conformational flexibility and enhance the inhibition by compounds that bind within the catalytic site.

### Maple syrup, sap and aqueous maple wood extracts inhibit *Listeria monocytogenes* SrtA *in vitro*

**Figure 7:**
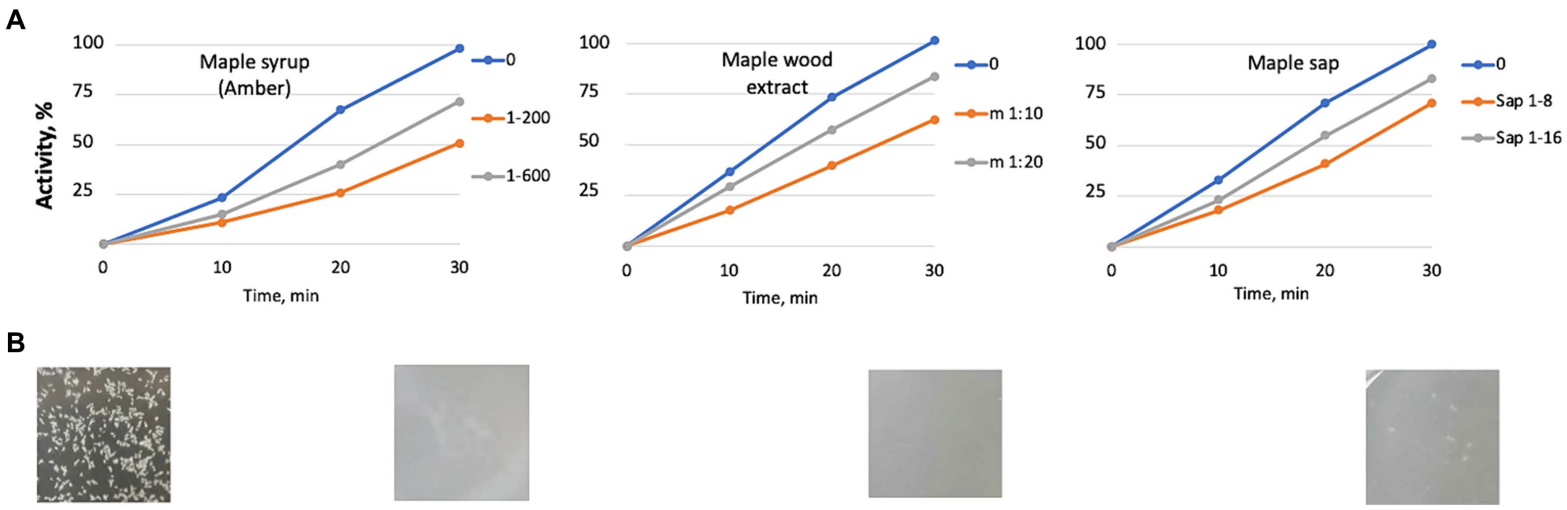
Maple products inhibiting cell aggregation in *L. monocytogenes* possess the anti-SrtA activity *in vitro*. **(A)** inhibition of the SrtA activity *in vitro*. Maple syrup at 1:200 and 1:600 dilutions; aqueous maple wood extract prepared as described in Elbakush et al. (2023); and maple sap. The averages of two experiments are displayed. Standard deviations did not exceed 17% of the average values. **(B)** Representative images of the appearance of bacterial cultures after 48-h incubation in the presence of the maple products.

We wanted to invvestigate whether diluted maple syrup exhibits SrtA inhibitory activity *in vitro*. A 1:200 dilution of Amber syrup inhibited SrtA activity by approximately 50% (Figure 7A). We were unable to test less diluted solutions due to high autofluorescence from the compounds in the maple syrup, which interfered with the fluorescence-based activity assay. Further, aqueous extracts from maple wood and diluted maple sap also demonstrated anti-SrtA activity (Figures 7A,B). These findings support the conclusion that SrtA inhibition is a key factor in the antibiofilm activity of aqueous maple wood extracts against *L. monocytogenes*.

## Discussion

Washing fresh fruits and vegetables and cleaning and disinfecting produce processing equipment might not always be sufficient for removing *L. monocytogenes* biofilms or preventing their formation (Oloketuyi and Khan, 2017; Fagerlund et al., 2020). Therefore, using safe and affordable natural products that inhibit biofilm formation and promote biofilm dispersion may be necessary. In our previous work, we identified aqueous extracts from maple, hickory, pecan, and star jasmine wood as potent antibiofilm agents against *L. monocytogenes* EPS-biofilms (Fulano et al., 2023). Producing these extracts from wood byproducts like shavings, chips, sawdust, and bark is cost-effective, particularly where wood is locally available. Additionally, the maple syrup industry represents a large source of antibiofilm compounds, with approximately one billion gallons of maple sap collected annually in Canada and the USA (Statistics Canada, 2023; USDA and National Agricultural Statistics Service, 2023). Given the long history of safe human consumption of maple sap and syrup, using maple wood-based products to prevent listerial biofilms seems promising.

In this study, we deciphered the mechanism of action of maple lignans, NTG and LR, and identified several new antibiofilm compounds. We show that maple wood extracts, including commercially available maple syrup, contain a cocktail of antibiofilm compounds. These compounds do not affect the synthesis or hydrolysis of Pss EPS (Figure 2) but inhibit its attachment to bacterial cells by targeting sortase A (SrtA) (Figure 3). We hypothesize that this effect is due to interference with the SrtA-mediated peptidoglycan anchoring of the Pss-specific lectin, which remains to be identified. Inhibiting SrtA causes the detachment of the lectin and Pss EPS from bacterial cells, reducing bacterial adherence to fresh produce and preventing clumping.

This mechanism of SrtA inhibition explains several observations that alternative antibiofilm mechanisms cannot. For example, maple compounds inhibit listeria attachment not only in Pss-overproducing strains but also in strains that do not synthesize Pss (Elbakush et al., 2023). This suggests that SrtA inhibition affects not only the Pss-lectin but also other surface proteins involved in attachment. In strain EGD-e, such proteins are not yet characterized, but in a related strain, 1040S, the *Listeria* cellulose-binding protein (Lcp) has been identified (Bae et al., 2013). Lcp homolog Lmo0842, encoded in the EGD-e genome, likely serves the same function. Since Lcp/Lmo0842 has the LPxTG sequence characteristic of SrtA targets, its anchoring to the peptidoglycan must be impairred by SrtA inhibition. This hypothesis is currently being tested.

Another observation supporting the SrtA inhibition mechanism is the relatively slow rate at which preformed Pss EPS-biofilms are dispersed by maple compounds. As shown in Figure 2C, diluted maple syrup causes an immediate but modest dispersion of listerial clumps, with no further increase over a 30-min period. This suggests that SrtA-dependent dispersion affects only newly synthesized EPS, while already anchored EPS is less affected. The rapid, modest clump dispersion also indicates an additional, SrtA-independent antibiofilm activity of maple compounds.

In addition to previously reported lignans NTG and LR (Elbakush et al., 2023), we found that ECG, IS, and its isomer scopoletin, also possess antibiofilm activity and act as potent SrtA inhibitors. Further, the common phytohormone AA, abundant in maple syrup, has moderate SrtA inhibitory activity (Figures 4A–E). Molecular modelling of SrtA interactions with these inhibitors showed that, despite structural differences, all bind within the catalytic site with negative ΔG values (Figure 6). Interestingly, binding modalities vary. ECG, the most potent SrtA inhibitor *in vitro* (Figure 4D) and *in vivo* (Figures 5A,B), primarily forms hydrogen bonds with the SrtA catalytic triad, while IS, the second most potent inhibitor, forms hydrophobic interactions with non-catalytic residues (Figures 6C,D). We found that the correlation between ΔG values and activity for other compounds is not straightforward. For example, previously described SrtA inhibitors, such as genistin (Liu et al., 2023), chalcone (Li et al., 2016), and baicalein (Lu et al., 2019), as well as inhibitors of SrtA from related pathogens, did not show antibiofilm activity (Supplementary Figure S2). This suggests that factors like extracellular matrix sequestration, poor solubility, or inactivation by media components might limit the effectiveness of these compounds.

Our work suggests that the antibiofilm potency of maple extracts is due to a cumulative effect of SrtA inhibitors (Figure 7). In the limited pairwise testings of SrtA inhibitors, we observed additive, but not synergistic, effects. However, other combinations of SrtA inhibitors or inhibitors with non-inhibitory maple compounds might yield synergistic effects. Some SrtA inhibitors may also bind to sites outside the catalytic pocket (Supplementary Figure S3), potentially enhancing inhibition by stabilizing SrtA in an inactive conformation.

It is worth noting that maple compounds are ineffective against bacteria that lack SrtA, like *S. typhimurium* (Figure 1), which support the idea that SrtA is the primary target. Sortases are attractive targets for antibacterial agents because they anchor cell wall adhesins involved in interactions with surfaces and cells, and their extracellular localization makes them accessible. Importantly, *srtA* mutants in *Listeria*, and also in *Streptococcus* and *Staphylococcus*, show reduced virulence (Schneewind and Missiakas, 2014). If maple products can inhibit sortases A in all pathogens from the Bacillota phylum, their utility may extend beyond combating *L. monocytogenes* biofilms.

## Materials and methods

### Bacterial strains, plasmids and growth conditions

The strains used in this study are listed in Table 2. The *L. monocytogenes* wild type strain, EGD-e, and its derivatives were grown in the liquid minimum HTM medium (Tsai and Hodgson, 2003) containing 3% glucose, HTM/G, at 30°C under shaking (220 rpm). For enumerating CFUs, cultures were plated onto Brain Heart Infusion (BHI) agar (Millipore Sigma) and incubated at 37°C for 24 h. The *Salmonella enterica* subsp. Typhimurium strains were also grown in HTM/G for biofilm experiments with pieces of fresh produce, and in Luria–Bertani (LB) for biofilm experiments in microtiter plates.

**Table 2:** Strains and plasmids used in this study.

Strains and plasmids	Relevant genotype or description	Reference or source
**Strains**		
*Escherichia coli*		
DH10β	Strain used for plasmid maintenance	New England BioLabs
*Listeria monocytogenes*		
EGD-e	Wild type	ATCC BAA-679^†^
*ΔpdeB/C/D*	EGD-e containing deletions in the *pdeB, pdeC* and *pdeD* genes	Chen et al. (2014)
*ΔpdeB/C/D ΔpssC*	*ΔpdeB/C/D* and in-frame deletion in *pssC*	Köseoğlu et al. (2015)
*ΔpdeB/C/D ΔsrtA*	*ΔpdeB/C/D* and in-frame deletion in *srtA*	This work
*ΔpdeB/C/D ΔsrtA*::pIMK2-*srtA*	Complementation of the *ΔsrtA* mutation by the chromosome-integrated wild-type *srtA* gene	This work
*Salmonella enterica* subsp. Typhimurium		
UMR1	Wild-type (a rdar_28C_ positive colony of ATCC14028) Nal^r^	Zogaj et al. (2001)
MAE97	High c-di-GMP strain overexpressing EPS. *UMR1 PcsgD1 ΔcsgBA102*	Römling et al. (2000)
**Plasmids**		
pET23a	Plasmid for His_6_-tagged protein overexpression	Invitrogen
pET23a-*srtA*	pET23a::*srtA*-His_6_	This study
pLR16-pheS*	Suicide vector for allelic exchange in *L. monocytogenes*	Argov et al. (2017)
pLR16-pheS*-Δ*srtA*	Plasmid for the in-frame *srtA* deletion	This study
pIMK2	Integration vector in *L. monocytogenes*	Monk et al. (2008)

^†^ATCC, American Type Culture Collection.

### Maple wood products and phytochemicals

Maple syrup (Amber grade) from Crown Maple (NY, United States) was diluted with sterile water and used at the indicated final dilutions, usually 1 part to 200 parts (1:200) of HTM/G medium or reaction buffer. Phytochemicals were purchased from the following suppliers: (−)-epicatechin gallate (ECG) from Aobius Inc. (MA, United States), nortrachelogenin-8’-O-β-D-glucopyranoside, lariciresinol, (+)-abscisic acid, scopoletin and isoscopoletin from Targetmol (MA, United States).

### Construction and complementation of the *srtA* mutant

The *srtA* gene was deleted by using the suicide vector for allelic exchange, pLR16-pheS* (Argov et al., 2017). Approximately 800-bp fragments upstream and downstream from the deleted regions of the *srtA* gene were separately amplified by PCR using primers pairs designated A and B, and C and D (Supplementary Table S1). The pLR16-pheS* plasmid was linearized with XhoI and KnpI and the two PCR fragments were cloned by Gibson assembly (NEB), and transformed into *Escherichia coli* NEB 10β. The pLR16-pheS*-*ΔsrtA* plasmid was purified, sequenced and transformed in the *L. monocytogenes* high c-di-GMP strain, *ΔpdeB/C/D*. Two transformants were grown at 41°C in the presence of 10 μg chloramphenicol (Cm) mL^−1^ to ensure integration of the plasmid into the genome via homologous recombination, and plated on BHI agar containing Cm at 41°C overnight. Five colonies were picked, grown at 37°C overnight with no antibiotics and plated on BHI agar. Colonies were screened on BHI agar supplemented with 18 mM fenclonine (MedChemExpress), which selects against the inserted plasmid. In-frame *srtA* deletion mutants were identified by colony-PCR. To complement the *ΔsrtA* mutation, the wild-type *srtA* gene, including its promoter, was cloned into the integrative vector pIMK2 (Monk et al., 2008). The primers used in this study are shown in Supplementary Table S1. The pIMK2-*srtA* plasmid was transformed in the *ΔpdeB/C/D ΔsrtA* strain. The chromosomal integrants were identified by kanamycin resistance. The restoration of the *srtA* gene was verified by PCR-amplification of the *srtA* gene fragment from the inserted pIMK2 and sequencing.

### Preparation and inoculation of pieces of fresh produce

Overnight *S. typhimurium* cultures were diluted 1:100 into 10 mL HTM/G medium in 125-mL flasks and grown at 30°C until optical density, A_600_, ~ 0.4, at which point pieces of fresh produce were added, and cultivation was continued for 48 h. Cantaloupe coupons (20 mm diameter × 4 mm thickness) were obtained by using a cork borer. Celery strands of approximately the same diameter was cut in pieces of ~20 mm in length. Preparation of the sterile cantaloupe and celery pieces was done as described earlier (Fulano et al., 2023). Following incubation for 48 h, produce pieces were aseptically withdrawn, rinsed in HTM/G twice to remove loosely bound biofilms, and mechanically macerated in the homogenizer (Stomacher^®^ 80 Biomaster, Seward, UK), as described earlier (Fulano et al., 2023). The serial dilutions of the homogenates were plated onto BHI agar plates for CFU enumeration after 24 h.

### RNA purification and quantitative RT-PCR, qRT-PCR

Total RNA was extracted by using Quick-RNA Fungal/Bacterial Miniprep Kit (Zymo Research, R2014) as instructed by the manufacturer. High c-di-GMP listerial strains were grown in 10 mL HTM/G medium with and without 1:200 diluted maple syrup for 48 h at 30°C. After brief centrifugation, cell pellets were resuspended in the RNA lysis buffer and disrupted mechanically with a BEAD BUG cell disrupter (Benchmark Scientific, 50 s, speed 400). Following RNA extraction, Turbo DNA-free kit (Invitrogen) was used to remove remaining chromosomal DNA. One μg of purified DNA-free RNA was converted to cDNA by using the iScript cDNA synthesis kit (Bio-Rad). qRT-PCR was performed by using IQ SYBR green Supermix (Bio-Rad). The mRNA levels of the *pssZ* gene and *srtA* genes were normalized to the mRNA level of *rpoB* used as a reference transcript (Supplementary Table S2).

### Proteins overexpression and purification

The *L. monocytogenes* PssZ protein was overexpressed in *E. coli* BL21(DE3) containing the overexpression plasmid, pET23a::*pssZ*-His_6_, as described previously (Köseoğlu et al., 2015). The DNA fragment encoding the *L. monocytogenes* SrtA protein lacking the first 70 amino acids was PCR-amplified from the genomic DNA using primers specified in Supplementary Table S1. The amplified fragment was digested with BamHI and NdeI and cloned into the pET23a vector. The recombinant protein containing the C-terminal His_6_-tag was overexpressed in *E. coli* BL21(DE3). Cultures of BL21(DE3) harboring the pET23a::*srtA*-His_6_ were grown in LB medium supplemented with ampicillin (100 mg/L) at 37°C with shaking until the absorbance reached an A_600_, 0.6–0.8. IPTG was then added to a final concentration of 1 mM, and the culture was grown for a further 12 h at room temperature. The cells were collected by centrifugation at 4°C, and the pellets were resuspended in the buffer containing 300 mM NaCl, 50 mM NaH_2_PO_4_, 10 mM imidazole, and protease inhibitors (APExBIO) (pH 7.4). The cells were disrupted by using a French press minicell (Spectronic Instruments, NJ). The crude cell extracts were centrifuged at 15,000 × g for 10 min. Soluble protein fractions were collected, mixed with pre-equilibrated Co^2+^ charger resin (TALON-metal affinity resin; TaKaRa) for 3 h at 4°C, and then placed into a column and extensively washed with resuspension buffer containing 25 mM imidazole. The SrtA::His_6_ protein was subsequently eluted by using 250 mM imidazole. Protein purity was assessed by SDS-PAGE, and the protein concentration was determined by using a Bradford protein assay (BIO-RAD).

### SrtA activity assays

SrtA activity was measured following previously described protocol (Li et al., 2016). Reactions (120 μL) were performed in the buffer (50 mM Tris–HCl, 150 mM NaCl, and 5 mM CaCl_2_; pH 8) containing 24 μg SrtA::His_6_ and 1 μg (8 nM) substrate, Dabcyl-LPETG-Edans (AnaSpec). Quantification of fluorometric intensity (350 nm excitation, 520 nm emission) was done using a microplate reader (SYNERGY H4, BioTek).

### Statistical analysis

Microsoft Excel was used for data processing and analysis. The bar charts display a mean ± standard deviation from three-to-five independent experiments, each performed in at least two replicates. Unpaired Student’s t-tests were performed using Prism 9 for Mac (GraphPad). The sortase activity measurements were performed in duplicate, and the averages are displayed.

## Data Availability

The raw data supporting the conclusions of this article will be made available by the authors, without undue reservation. Requests to access the datasets should be directed to Mark Gomelsky, gomelsky@uwyo.edu.
