# Supplementary material for: Maple compounds prevent biofilm formation in Listeria monocytogenes via sortase inhibition
Source: Front Microbiol. 2024 Sep 16;15:1436476. doi: 10.3389/fmicb.2024.1436476 (PMC11439720; doi:10.3389/fmicb.2024.1436476)

**Supporting information for**

**Maple compounds prevent biofilm formation in *Listeria monocytogenes* via sortase inhibition**

Ahmed M. Elbakush, Oliver Trunschke, Sulman Shafeeq, Ute Römling and Mark Gomelsky

**Table of contents**

Suppl. Table 1: Primers are used in this study.

Suppl. Table 2. qRT-PCR primers.

Suppl. Fig. 1. Effect of aqueous maple extract on biofilm formation of *S.* Typhimurium in microtiter plates.

Suppl. Fig. 2. *In vitro* SrtA inhibitors do not necessarily inhibit EPS-biofilms in *L. monocytogenes*.

Suppl. Fig. 3. Molecular modeling of the *L. monocytogenes* SrtA interactions with selective maple compounds.

**Suppl. Table 1. Primers are used in this study.**

| **Primer name** | **Sequence** | **Purpose** |
| --- | --- | --- |
| srtA probe A | AAGGGAACAAAAGCTGGTACACCAAATGCATCGCCAAG | In-frame *srtA* deletion |
| srtA probe B | TTTTTCCTCAATGATTCCTCCTTGTAACTATTTC |  |
| srtA probe C | GAGGAATCATTGAGGAAAAAAAGACAGC |  |
| srtA probe D | GCTTATCGATACCGTCGACCAGAAAGTTACTTTAAACGGC |  |
| srtA:His F | GAAGGAGATATACAACATATGGCGGCAAATTACGAC | *srtA* overexpression |
| srtA:His R | CGGAGCTCGAATTCGTTTACTAGGGAAATATTTATTCTC |  |
| P-srtA F | GTG AAA CCC ATG GAA AAG CTT TAC AAT TTT GTC ACA GG | *ΔsrtA*  complementation |
| P-srtA R | CCG GGC CCC CCC TCG ATT ATT TAC TAG GGA AAT ATT TAT TC |  |

**Suppl. Table 2. qRT-PCR primers.**

| **Primer name** | **Sequence** | **Source** |
| --- | --- | --- |
| rpoB F | GAAGTTTTGCGCGAATCAGTG | This study |
| rpoB R | CAGTGGAGCCGCATAGTTTG | This study |
| pssZ F | CAAACCATTAACGCCGATCCA | This study |
| pssZ R | TCCCTTTGGAAGCTAGTTCAGT | This study |
| srtA F | TCCGCTAGCAGGTCACCATA | This study |
| srtA R | CAGCGTTATTCTAGCATCTTTCGT | This study |

**Suppl. Fig. 1.** **Effect of aqueous maple extract on biofilm formation of *S.* Typhimurium in microtiter plates.** *S.* Typhimurium UMR1 rdar_28_^+^ biofilm forming variant was incubated in LB supplemented with diluted maple wood extract for 24 h at 28 °C in 96 well plates. The wells were rinsed with water and stained with a 0.2 % Crystal violet solution. The well-attached biofilms were quantified as concentrations of Crystal violet (A_595_) following its dissolution, as described earlier (Zogaj et al., 2001). Shown is one of at least three representative experiments conducted with six technical replicates each. Aqueous maple extract was obtained by soaking maple wood chips in water (1 g per 10 mL), as described earlier (Fulano *et al*., 2023).

**Suppl. Fig. 2. *In vitro* SrtA inhibitors do not necessarily inhibit EPS-biofilms in *L. monocytogenes*. A,** *L. monocytogenes* SrtA inhibitors, genistin, baicalein, and chalcone are ineffective as anti-EPS agents. **B,** The inhibitors of SrtA from the related Bacillota pathogens, astibin, curcumin, and morin are also ineffective. The compounds were tested at 60 and 120 μM (displayed) concentrations. Chalcone solution (dissolved in DMSO) precipitates when mixed with the HTM/G growth medium.

**­Suppl. Fig. 3. Molecular modeling of the *L. monocytogenes* SrtA interactions with selective maple compounds.** The X-ray structure of the *L. monocytogenes* SrtA (PDB: 5HU4) was used to predict binding modes of the maple compounds using the Autodoc Vina software (Trott and Olson, 2010). The predicted binding sites and chemical bonds are shown on the panels corresponding to individual compounds. Grid Box measurements were established with AutoDockTools-1.5.7. Box coordinates: X: 5.117Å, Y: 20.439Å , Z: 11.52Å ; Box Size: X: 52Å, Y: 58Å, Z: 58Å, with default spacing 0.375, Mode Number: 3, Energy Range: 5, Exhaustiveness: 8. The amino acid residues of the catalytic triad of SrtA (H127, C188, R197) are colored in magenta. H-bonds are shown in red. Amino acids forming hydrophobic interactions with maple compounds are shown in yellow, except for hydrophobic interactions with the amino acids of the catalytic triad, which are shown in black. Inhibitors: **A**, LR; **B**, IS; **C**, ECG; **D**, AA.


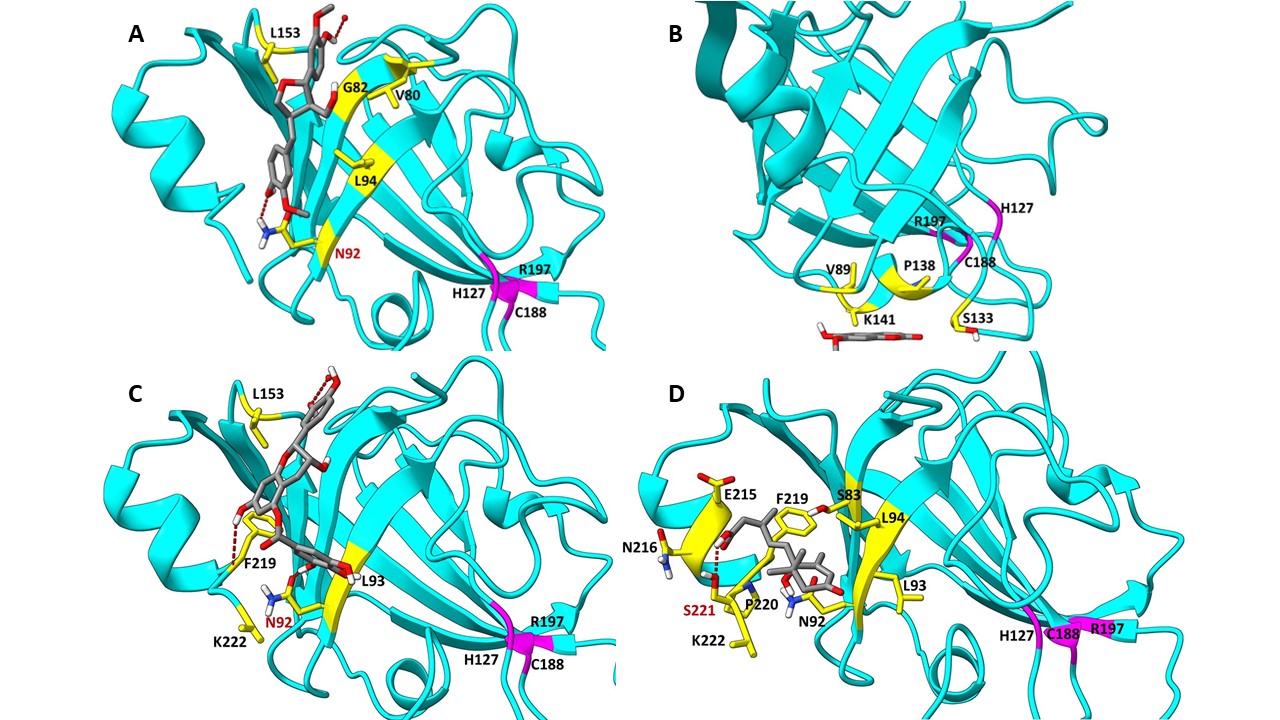

Supplement: Supplementary file 1 [file Data_Sheet_1.docx]
